# Supplementary material for: Modeling glioblastoma heterogeneity as a dynamic network of cell states
Source: Mol Syst Biol. 2021 Sep 16;17(9):e10105. doi: 10.15252/msb.202010105 (PMC8444284; doi:10.15252/msb.202010105)
Supplement: Supplementary file 5 — Source Data for Figure 3 [file MSB-17-e10105-s001.zip › Figure3A_sourcedata/GSEA_3065/hallmarks_state4.GseaPreranked.1623416296068/HALLMARK_INTERFERON_GAMMA_RESPONSE.html]

Details for gene set HALLMARK\_INTERFERON\_GAMMA\_RESPONSE[GSEA]

|  || Dataset | state4 |
| Phenotype | NoPhenotypeAvailable |
| Upregulated in class | na\_pos |
| GeneSet | HALLMARK\_INTERFERON\_GAMMA\_RESPONSE |
| Enrichment Score (ES) | 0.27411908 |
| Normalized Enrichment Score (NES) | 1.0552695 |
| Nominal p-value | 0.36131388 |
| FDR q-value | 0.4119759 |
| FWER p-Value | 0.995 |
Table: GSEA Results Summary

  

Fig 1: Enrichment plot: HALLMARK\_INTERFERON\_GAMMA\_RESPONSE      
 Profile of the Running ES Score & Positions of GeneSet Members on the Rank Ordered List

  

| PROBE | GENE SYMBOL | GENE\_TITLE | RANK IN GENE LIST | RANK METRIC SCORE | RUNNING ES | CORE ENRICHMENT || 1 | CD74 |  |  | 64 | 0.281 | 0.0447 | Yes |
| 2 | TXNIP |  |  | 92 | 0.254 | 0.0901 | Yes |
| 3 | BPGM |  |  | 223 | 0.193 | 0.1068 | Yes |
| 4 | CDKN1A |  |  | 319 | 0.171 | 0.1250 | Yes |
| 5 | SRI |  |  | 458 | 0.143 | 0.1306 | Yes |
| 6 | IFITM3 |  |  | 546 | 0.132 | 0.1423 | Yes |
| 7 | UBE2L6 |  |  | 609 | 0.125 | 0.1567 | Yes |
| 8 | MVP |  |  | 630 | 0.122 | 0.1774 | Yes |
| 9 | SOCS3 |  |  | 701 | 0.114 | 0.1884 | Yes |
| 10 | VAMP5 |  |  | 750 | 0.108 | 0.2018 | Yes |
| 11 | C1R |  |  | 778 | 0.106 | 0.2181 | Yes |
| 12 | STAT2 |  |  | 824 | 0.102 | 0.2308 | Yes |
| 13 | IFITM2 |  |  | 876 | 0.098 | 0.2417 | Yes |
| 14 | ISG15 |  |  | 884 | 0.097 | 0.2595 | Yes |
| 15 | BST2 |  |  | 953 | 0.092 | 0.2665 | Yes |
| 16 | SSPN |  |  | 1013 | 0.087 | 0.2741 | Yes |
| 17 | PSME1 |  |  | 1273 | 0.072 | 0.2464 | No |
| 18 | ARID5B |  |  | 1327 | 0.070 | 0.2515 | No |
| 19 | MT2A |  |  | 1542 | 0.060 | 0.2288 | No |
| 20 | LAP3 |  |  | 1570 | 0.059 | 0.2360 | No |
| 21 | ZNFX1 |  |  | 1913 | 0.049 | 0.1905 | No |
| 22 | SAMD9L |  |  | 1929 | 0.049 | 0.1976 | No |
| 23 | STAT1 |  |  | 1969 | 0.048 | 0.2006 | No |
| 24 | IFI35 |  |  | 2001 | 0.047 | 0.2048 | No |
| 25 | TDRD7 |  |  | 2382 | 0.037 | 0.1508 | No |
| 26 | RIPK1 |  |  | 2510 | 0.035 | 0.1371 | No |
| 27 | IRF7 |  |  | 2713 | 0.030 | 0.1104 | No |
| 28 | MYD88 |  |  | 2736 | 0.030 | 0.1127 | No |
| 29 | RIPK2 |  |  | 2812 | 0.028 | 0.1062 | No |
| 30 | RNF213 |  |  | 2878 | 0.027 | 0.1010 | No |
| 31 | NAMPT |  |  | 3040 | 0.024 | 0.0797 | No |
| 32 | PSMB9 |  |  | 3055 | 0.024 | 0.0822 | No |
| 33 | PELI1 |  |  | 3130 | 0.022 | 0.0746 | No |
| 34 | SP110 |  |  | 3144 | 0.022 | 0.0769 | No |
| 35 | CIITA |  |  | 3406 | 0.015 | 0.0378 | No |
| 36 | LYSMD2 |  |  | 3554 | 0.011 | 0.0162 | No |
| 37 | USP18 |  |  | 3580 | 0.010 | 0.0140 | No |
| 38 | LGALS3BP |  |  | 3757 | 0.003 | -0.0138 | No |
| 39 | STAT3 |  |  | 3937 | -0.006 | -0.0416 | No |
| 40 | PARP14 |  |  | 3950 | -0.007 | -0.0422 | No |
| 41 | PDE4B |  |  | 4063 | -0.013 | -0.0578 | No |
| 42 | BTG1 |  |  | 4064 | -0.013 | -0.0552 | No |
| 43 | TAPBP |  |  | 4109 | -0.016 | -0.0592 | No |
| 44 | EIF2AK2 |  |  | 4256 | -0.025 | -0.0777 | No |
| 45 | PSMA2 |  |  | 4300 | -0.029 | -0.0791 | No |
| 46 | ISG20 |  |  | 4361 | -0.033 | -0.0823 | No |
| 47 | ADAR |  |  | 4453 | -0.040 | -0.0891 | No |
| 48 | ISOC1 |  |  | 4505 | -0.045 | -0.0886 | No |
| 49 | PSMB2 |  |  | 4557 | -0.048 | -0.0873 | No |
| 50 | OAS3 |  |  | 4800 | -0.068 | -0.1131 | No |
| 51 | PSMA3 |  |  | 4817 | -0.069 | -0.1022 | No |
| 52 | ARL4A |  |  | 4864 | -0.073 | -0.0954 | No |
| 53 | AUTS2 |  |  | 4992 | -0.082 | -0.0999 | No |
| 54 | IRF1 |  |  | 5157 | -0.098 | -0.1072 | No |
| 55 | LY6E |  |  | 5230 | -0.104 | -0.0985 | No |
| 56 | MTHFD2 |  |  | 5256 | -0.107 | -0.0817 | No |
| 57 | SPPL2A |  |  | 5336 | -0.112 | -0.0725 | No |
| 58 | METTL7B |  |  | 5354 | -0.114 | -0.0530 | No |
| 59 | PSME2 |  |  | 5418 | -0.121 | -0.0395 | No |
| 60 | PFKP |  |  | 5471 | -0.128 | -0.0229 | No |
| 61 | PNPT1 |  |  | 5531 | -0.135 | -0.0061 | No |
| 62 | B2M |  |  | 5823 | -0.188 | -0.0162 | No |
| 63 | TNFAIP6 |  |  | 5872 | -0.199 | 0.0150 | No |
| 64 | UPP1 |  |  | 6016 | -0.245 | 0.0398 | No |
Table: GSEA details [plain text format]

  

Fig 2: HALLMARK\_INTERFERON\_GAMMA\_RESPONSE: Random ES distribution      
 Gene set null distribution of ES for **HALLMARK\_INTERFERON\_GAMMA\_RESPONSE**
